# Role of p16 testing in cervical cancer screening among HIV-infected women

**DOI:** 10.1371/journal.pone.0185597

**Published:** 2017-10-12

**Authors:** Christine J. McGrath, Rochelle Garcia, Trong T. Trinh, Barbra A. Richardson, Grace C. John-Stewart, Evans Nyongesa-Malava, Nelly R. Mugo, Emily H. Glynn, Samah R. Sakr, Hugo De Vuyst, Michael H. Chung

**Affiliations:** 1 Department of Global Health, University of Washington, Seattle, Washington United States of America; 2 Department of Pathology, University of Washington, Seattle, Washington, United States of America; 3 Department of Biostatistics, University of Washington, Seattle, Washington, United States of America; 4 Division of Vaccine and Infectious Diseases, Fred Hutchinson Cancer Research Center, Seattle, Washington, United States of America; 5 Department of Medicine, University of Washington, Seattle, Washington, United States of America; 6 Department of Pediatrics, University of Washington, Seattle, Washington, United States of America; 7 Department of Epidemiology, University of Washington, Seattle, Washington, United States of America; 8 Kenyatta National Hospital, Nairobi, Kenya; 9 Coptic Hospital, Nairobi, Kenya; 10 International Agency for Research on Cancer, Lyon, France; Georgetown University, UNITED STATES

## Abstract

**Background:**

p16 immunohistochemistry is used to evaluate for HPV-associated cervical intraepithelial neoplasia. The diagnostic performance of p16 in HIV infection is unclear.

**Methods:**

Between June-December 2009, HIV-infected women underwent Papanicolaou (Pap) smear, human papillomavirus (HPV) testing, visual inspection with acetic acid (VIA), and colposcopy-directed biopsy as the disease gold standard at a HIV clinic in Kenya. Pap smears were evaluated for p16 expression. Sensitivity, specificity, positive predictive value (PPV), and area under the receiver operating characteristic curve (AUC) of p16 to detect CIN2/3 on histology and the impact of immunosuppression and ART was assessed.

**Results:**

Of 331 cervical samples with p16 expression, p16 sensitivity and specificity to detect CIN2/3 was 54.1% and 72.4% respectively, which was lower than Pap and HPV in sensitivity, but higher in specificity than Pap, HPV, and VIA. Combining tests and p16 reduced sensitivity and increased specificity of Pap from 90.5% to 48.7% and 51.4% to 81.7%; of VIA from 59.5% to 37.8% and 67.6% to 89.9%; and of HPV from 82.4% to 50.0% and 55.3% to 84.8%. Combination p16 increased the PPV of Pap from 34.9% to 43.4%; of HPV from 34.7% to 48.7%; and VIA from 34.9% to 51.9%. Adjunctive p16 did not change AUC (P>0.05). P16 performance was not altered by immunosuppression or ART use. Combining p16 with HPV and VIA reduced the variation in HPV and VIA performance associated with CD4 and ART.

**Conclusion:**

As an adjunctive test in HIV-infected women, p16 immunohistochemistry increased specificity and PPV of HPV and VIA for CIN2/3, and was not altered in performance by immunosuppression, ART, or age.

## Introduction

Cervical cancer is one of the most prevalent cancers worldwide with the greatest burden among women in resource-limited settings [1]. Cervical neoplasm screening methods including Papanicolaou (Pap) smear, human papillomavirus (HPV) testing, and visual inspection with acetic acid (VIA) have dramatically reduced cervical cancer incidence and mortality [2, 3]. However, in resource-limited settings, cervical neoplasm screening is not routine and standard screening tools are not widely available [4]. Compared to HIV-uninfected women, the performance of existing cervical neoplasm screening methods may be less effective among HIV-infected women [5, 6].

HIV-infected women are disproportionately affected by HPV and at increased risk of developing HPV-associated cervical neoplasm and invasive cervical carcinoma [7, 8]. While HPV sensitivity has been shown to be high among HIV-infected women, HPV specificity is limited (55.7%) with further reductions associated with younger age, advanced immunosuppression, and shorter duration of antiretroviral therapy (ART) [5]. Similarly, VIA sensitivity was lower among older women and specificity varied by ART use [5]. Due to the elevated risk of cervical neoplasm in HIV-infected women, alternative screening strategies including biomarkers for cervical cancer precursors/intraepithelial may improve early detection and prevent invasive cervical carcinoma.

In HIV-uninfected women, p16 protein expression has been shown to distinguish mild cervical intraepithelial neoplasm (CIN1) from moderate to severe intraepithelial neoplasm (CIN2/3) with similar sensitivity but higher specificity for CIN2/3 than HPV testing [9, 10]. Moreover, the sensitivity and specificity of p16 in identifying intraepithelial neoplasia has been shown to be as high as 90% and 80%, respectively, in the general population [11–13]. There is limited data on the performance of p16 testing among HIV-infected women. An association between HIV infection and reduced p16 expression in CIN2/3 has been reported [14].

The objective of this study was to determine the performance of p16 immunohistochemistry to detect CIN2/3 among HIV-infected women while assessing the effect of immune status, ART use, and age, and to evaluate the utility of p16 staining alone and in combination with Pap smear, HPV, and VIA.

## Methods

### Study population and procedures

A total of 500 HIV-infected women were recruited from the Coptic Hope Center for Infectious Diseases in Nairobi, Kenya between June-December 2009. HIV-infected women were eligible for cervical screening if they were between ages 18 and 55 years, had an intact cervix, and never received treatment for cancerous or pre-cancerous cervical lesions. Methodology has been described elsewhere [5]. Demographic and clinical information was collected using standardized questionnaires and participants underwent pelvic examination and CD4 cell count. HIV history and ART use were abstracted from medical records. Women underwent Pap smear and an endocervical brush collected for HPV. VIA was performed followed by colposcopy-directed biopsy. Pap and biopsy results were determined using Bethesda 1991 revised classification scheme and Richart CIN staging [15, 16]. Colposcopy-directed biopsy and its histology results were used as the gold standard for diagnosis.

Written informed consent was obtained from all study participants. Ethical approval was received by the University of Washington, Kenyatta National Hospital, and International Agency for Research on Cancer.

### Laboratory methods

After Pap smears were read for cytology, slides were stored until shipment to Seattle, Washington to undergo p16 immunohistochemistry. After removing coverslips, the CINtec® histology kit (Ventana Medical Systems, Inc. USA) was used to detect and stain for qualitative expression of p16 protein per manufacturer’s instructions [17]. Positive p16 protein was defined as the presence of any staining as compared to the absence of staining or p16 negative. HPV DNA typing was performed using GP5+/6+-mediated PCR with an enzyme immunoassay to detect 14 high-risk HPV types (16, 18, 31, 33, 35, 39, 45, 51, 52, 56, 58, 59, 66, 68). Positivity for any of these types was considered HPV positive [18].

### Statistical methods

Disease was defined as the detection of CIN2/3 by colposcopy-directed biopsy on all women. The sensitivity, specificity, positive predictive value (PPV), negative predictive value (NPV), and area under the receiver operating characteristic curve (AUC) of abnormal Pap smear, defined as atypical squamous cells of undetermined significance or greater (≥ASCUS), HPV, and positive VIA were compared to the positive detection of p16 protein alone and in combination with other screening tests. Combinations included Pap with p16, HPV with p16, and VIA with p16. In these combinations, a positive screening result was defined as both tests being positive.

Comparisons of sensitivity and specificity were stratified by CD4 count (≤350 and >350 cells/μl), ART (none, <2 years and ≥2 years), and age (<40 and ≥40 years) and compared using chi-square tests. Statistical analyses were performed using STATA 13 (Stata Corporation, College Station, Texas, USA).

## Results

### Study population

Of 500 women screened, 471 had histology results, of which 331 (70.3%) cytology samples had adequate p16 results, 117 (24.8%) had indeterminate p16 results, and 23 (6.9%) were missing. Of the 331 women with p16 results, median age was 38.0 years and 80.7% were between ages 30–49 years. Forty percent were married and 34.7% reported one lifetime sexual partner. At screening, the median CD4 count was 371 cells/μl [Interquartile range (IQR), 249–544], 70% had CD4 <500 cells/μl and 52.4% had been on ART ≥2 years. There was no difference in age, CD4 count, or ART use between subjects with known results and those with indeterminate p16 results.

### Cervical neoplasm screening

On histology, 125 (37.8%) were normal, 132 (39.9%) CIN1, 43 (13.0%) CIN2, and 31 (9.4%) CIN3. On Pap, 128 (38.7%) were normal, 56 (16.9%) ASCUS, 79 (23.9%) LSIL, 57 (17.2%) HSIL, and 11 (3.3%) indeterminate. On p16, 111 (33.5%) were positive; on Pap, 192 (58.0%) were positive; on HPV, 176 (53.2%) were positive; and on VIA, 126 (38.5%) were positive. There was no difference in HPV, Pap, and histology between subjects with known results and subjects with indeterminate p16 results.

### Sensitivity and specificity and associations with CD4 count, ART, and age

**Fig 1:**
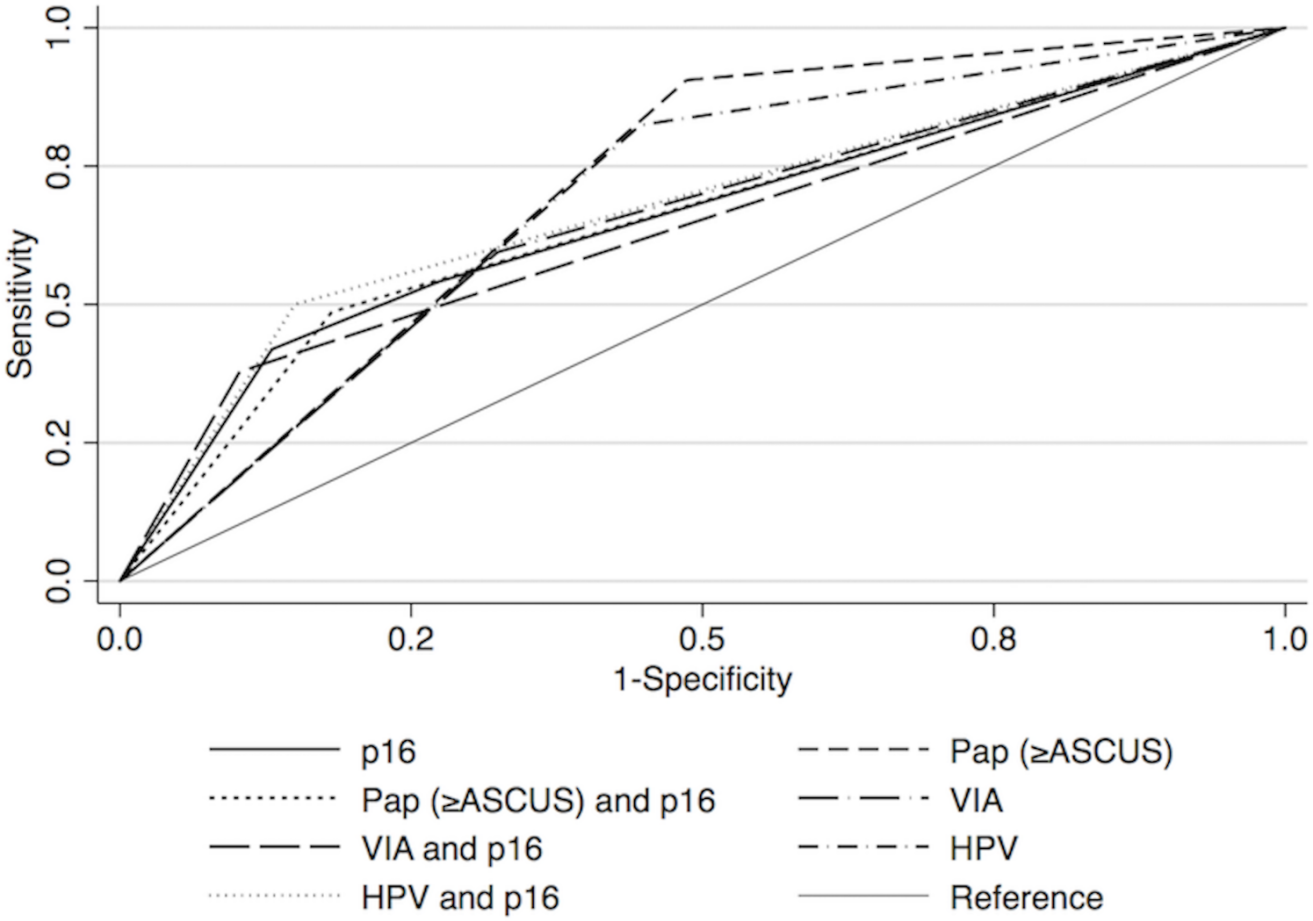
Receiver operating characteristic curves of screening methods individually and in combination with p16 for detection of CIN2/3 in HIV-infected women.

P16 sensitivity, specificity, PPV, NPV, and AUC were: 54.1%, 72.4%, 36.0%, 84.6%, and 0.63% (Table 1, Fig 1). Combining p16 with Pap reduced sensitivity and increased specificity and PPV from 90.5% to 48.7%, 51.4% to 81.7%, and 34.9% to 43.4%, respectively. Similarly, addition of p16 to HPV reduced sensitivity and increased specificity and PPV from 82.4% to 50.0%, 55.3% to 84.8%, and 34.7% to 48.7%. As an adjunct to VIA, p16 reduced sensitivity and increased specificity and PPV from 59.5% to 37.8%, 67.6% to 89.9%, and 33.9% to 51.9%. Combining tests with p16 did not significantly change the AUC (P>0.05 for each method).

**Table 1:** Sensitivity, specificity, positive predictive value (PPV), negative predictive value (NPV), and area under the receiver operating characteristic curve (AUC) of screening methods individually and in combination with p16 to detect CIN2/3 (n = 331).

	CIN 2/3	Sensitivity	Specificity	AUC	PPV	NPV
	(n = 74)	(95% CI)	(95% CI)	(95% CI)	(95% CI)	(95% CI)
p16						
Positive	40	54.1 (42.1–65.7)	72.4 (66.5–77.8)	0.63 (0.57–0.70)	36.0 (27.1–45.7)	84.6 (79.1–89.1)
Negative	34					
Pap (≥ASCUS)						
Positive	67	90.5 (81.5–96.1)	51.4 (45.1–57.6)	0.71 (0.66–0.76)	34.9 (28.2–42.1)	95.0 (89.9–98.0)
Negative	7					
Pap (≥ASCUS) and p16						
Positive	36	48.7 (36.9–60.6)	81.7 (76.4–86.2)	0.65 (0.59–0.71)	43.4 (32.5–54.7)	84.7 (79.6–88.9)
Negative	38					
VIA						
Positive	44	59.5 (47.4–70.7)	67.6 (61.4–73.3)	0.64 (0.57–0.70)	34.9 (26.6–43.9)	85.1 (79.4–89.7)
Negative	30					
VIA and p16						
Positive	28	37.8 (26.8–49.9)	89.9 (85.5–93.3)	0.64 (0.58–0.70)	51.9 (37.8–65.7)	83.4 (78.5–87.6)
Negative	46					
HPV						
Positive	61	82.4 (71.8–90.3)	55.3 (49.0–61.4)	0.69 (0.64–0.74)	34.7 (27.7–42.2)	91.6 (86.1–95.5)
Negative	13					
HPV and p16						
Positive	37	50.0 (38.1–61.9)	84.8 (79.8–89.0)	0.67 (0.61–0.74)	48.7 (37.0–60.4)	85.5 (80.6–89.6)
Negative	37					

P16 performance was not altered by immune status, ART duration, or age at screening (Table 2). Sensitivity and specificity of p16 testing was similar at CD4 ≤350 and >350 cells/μl (51.4% vs. 56.8% and 69.9% vs. 74.3%, respectively) and among women <40 years than ≥40 years (54.1% vs. 54.1% and 75.6% vs. 67.3%). While p16 sensitivity was higher among women on ART ≥2 years (62.2%) compared to women off ART (44.4%) and on ART <2 years (47.4%), it was not statistically significant (P = 0.37). Similarly, p16 specificity did not differ by ART ≥2 years compared to no ART and ART <2 years (71.3% vs. 75.0% vs. 70.8%, P = 0.83).

**Table 2:** Sensitivity and specificity of screening methods to detect CIN2/3 compared by CD4 count (A), ART duration (B), and age at screening (C) (n = 331).

Sensitivity	CD4 ≤350		CD4 >350		P-value
	n = 37 CIN2/3		n = 37 CIN2/3		
**A) CD4 count**					
p16	51.4		56.8		0.64
Pap (≥ASCUS)	91.9		89.2		0.69
Pap (≥ASCUS) and p16	54.1		43.2		0.35
VIA	64.9		54.1		0.34
VIA and p16	35.1		40.5		0.63
HPV	81.1		83.8		0.76
HPV and p16	43.2		56.8		0.25
Specificity	n = 113 ≤CIN1		n = 144 ≤CIN1		
p16	69.9		74.3		0.43
Pap (≥ASCUS)	48.7		53.5		0.45
Pap (≥ASCUS) and p16	80.5		82.6		0.66
VIA	64.9		69.7		0.41
VIA and p16	86.7		92.4		0.14
HPV	47.8		61.1		0.03
HPV and p16	82.3		86.8		0.32
Sensitivity	Off ART	On ART <2 years		On ART ≥2 years	P-value
	n = 18 CIN2/3	n = 19 CIN2/3		n = 37 CIN2/3	
**B) ART Duration**					
p16	44.4	47.4		62.2	0.37
Pap (≥ASCUS)	94.4	94.7		86.5	0.49
Pap (≥ASCUS) and p16	44.4	47.4		51.4	0.88
VIA	50.0	63.2		62.2	0.64
VIA and p16	33.3	31.6		43.2	0.63
HPV	88.9	78.9		81.1	0.70
HPV and p16	44.4	36.8		59.5	0.24
Specificity	n = 72 ≤CIN1	n = 48 ≤CIN1		n = 136 ≤CIN1	
p16	75.0	70.8		71.3	0.83
Pap (≥ASCUS)	52.8	45.8		52.2	0.71
Pap (≥ASCUS) and p16	83.3	79.2		81.6	0.85
VIA	63.9	51.1		75.2	0.007
VIA and p16	90.3	81.3		92.7	0.08
HPV	48.6	45.8		61.8	0.07
HPV and p16	84.7	81.3		86.0	0.73
Sensitivity	Age <40 years		Age ≥40 years		P-value
	n = 37 CIN2/3		n = 37 CIN2/3		
**C) Age**					
p16	54.1		54.1		1.00
Pap (≥ASCUS)	91.9		89.2		0.69
Pap (≥ASCUS) and p16	51.4		46.0		0.64
VIA	75.7		43.2		0.004
VIA and p16	45.9		29.7		0.15
HPV	78.4		86.5		0.36
HPV and p16	51.4		48.7		0.82
Specificity	n = 156 ≤CIN1		n = 101 ≤CIN1		
p16	75.6		67.3		0.15
Pap (≥ASCUS)	50.0		53.5		0.59
Pap (≥ASCUS) and p16	83.3		79.2		0.40
VIA	64.5		72.5		0.19
VIA and p16	89.1		91.1		0.61
HPV	48.1		66.3		0.004
HPV and p16	84.0		86.1		0.64

HPV specificity was higher at CD4 >350 than ≤350 cells/μl (61.1% vs. 47.8%, P = 0.03), however, addition of p16 to HPV reduced this difference (86.8% vs. 82.3%, P = 0.32). Combining p16 with HPV reduced the difference in HPV specificity associated with ART ≥2 years versus no ART and ART <2 years (61.8% vs. 48.6% and 45.8%, P = 0.07 for HPV and 86.0% vs. 84.7% and 81.3%, P = 0.73 for combination HPV and p16), and younger vs. older age (48.1% vs. 66.3%, P = 0.004 for HPV and 84.0% and 86.1%, P = 0.64 for combination HPV and p16). Adjunct p16 testing reduced the variability in VIA sensitivity associated with age (75.7% in <40 years vs. 43.2% ≥40 years, P = 0.004 for VIA and 45.9% vs. 29.7%, P = 0.15 for VIA and p16) and the differences in VIA specificity associated with ≥2 years ART vs. no ART and <2 years ART (75.2% vs. 63.9% and 51.1%, P = 0.007 for VIA and 92.7% vs. 90.3% and 81.3%, P = 0.08 for VIA and p16).

## Discussion

Among HIV-infected women, the use of p16 for the detection of histologically confirmed CIN2/3 had higher specificity (72.4%) and lower sensitivity (54.1%) compared to Pap, VIA, and HPV. While the performance of p16 alone was comparable to VIA, p16 sensitivity was markedly lower than HPV and Pap (≥ASCUS) and AUC was comparable between methods. As an adjunctive screening test for cervical neoplasia among HIV-infected women, p16 decreased sensitivity but increased specificity and PPV and its performance was not altered by immune status, ART duration, and age at screening.

P16 protein expression as a screening method and adjunct test among HIV-infected women is not well documented. Studies of p16 in HIV-negative women have shown sensitivity from 79–97%, specificity from 71–85%, and PPV from 41–91% [10, 19–21]. In our population of HIV-infected women, p16 sensitivity (54%), specificity (72%), and PPV (36%) were lower, irrespective of ART and immune status. This is in agreement with a study showing decreased p16 expression in HIV-infected women with CIN2/3 compared to their HIV-uninfected counterparts [14]. While our results do not support p16 testing alone, as an adjunctive test p16 increased the PPV of Pap, HPV, and VIA. Expression of p16 protein is associated with HPV integration and increases from no expression in normal tissue to overexpression in cervical intraepithelial neoplasia and carcinoma [22–24]. As a potential marker of progression, p16 positive lesions may therefore be important precancerous/intraepithelial neoplasms to treat. This is of particular relevance in settings where other triage methods including colposcopy directed-biopsy are less readily available.

The effect of immunosuppression and ART on the performance of cervical neoplasia tests in HIV-infected women has been documented [5, 6]. Unlike these other screening tests, the sensitivity and specificity of p16 was not influenced by HIV-associated immunosuppression, ART duration, or age. Moreover, adjunctive p16 testing removed the associations between HPV sensitivity and specificity and CD4 count and little or no ART. U.S. guidelines recommend HPV testing for primary cervical cancer screening followed by cytology or colposcopy among HPV-positive women [25]. In HIV-endemic settings, World Health Organization (WHO) recommendations include HPV testing followed by VIA to determine eligibility for treatment if HPV positive [26]. As HIV-infected women are at increased risk of HPV-associated disease, adjunctive p16 testing may help to discriminate between transient HPV infections and precancerous lesions/intraepithelial neoplasia, reducing overtreatment.

A major strength of this study is the use of colposcopy-directed biopsy on all women. As a result, our findings may better reflect p16 sensitivity in combination with other cervical neoplasia screening methods. In addition, this study included detailed data on CD4 count and ART exposure. However, this study has several limitations. P16 staining was performed on archived samples, of which 24.8% had indeterminate results. Sensitivity analysis showed that there was no difference between subjects with indeterminate and determinate p16 results by sociodemographic characteristics, clinical factors, or CIN2/3. The use of archived samples may have reduced quality due to long-term storage and the de-staining and re-staining procedures. Cell loss may have occurred during the removal of plastic coverslips, reducing p16 interpretability. Additional studies are needed to investigate p16 staining on cytology samples without prior staining. This study did not use positive p16 control slides to ensure internal quality assurance and this may limit interpretability of our results. Finally, while we had detailed data on immune status and ART duration, we did not have longitudinal data related to the duration of immunosuppression, HIV diagnosis date, and progression or persistence of cervical lesions/neoplasm.

As an adjunctive test to HPV and VIA in HIV-infected women, p16 increased specificity and PPV and reduced the variation in HPV and VIA performance associated with immunosuppression and ART duration. As WHO and U.S. guidelines recommend HPV genotyping as the primary screening for referral and treatment, adjunctive p16 testing may reduce unnecessary colposcopy and subsequent overtreatment among HIV-infected women.

## Supporting information

S1 File P16 Dataset. p16_PlosOne.xls.
(XLS)
